# Cancer Cell-Derived Extracellular Vesicles Are Associated with Coagulopathy Causing Ischemic Stroke via Tissue Factor-Independent Way: The OASIS-CANCER Study

**DOI:** 10.1371/journal.pone.0159170

**Published:** 2016-07-18

**Authors:** Oh Young Bang, Jong-Won Chung, Mi Ji Lee, Suk Jae Kim, Yeon Hee Cho, Gyeong-Moon Kim, Chin-Sang Chung, Kwang Ho Lee, Myung-Ju Ahn, Gyeong Joon Moon

**Affiliations:** 1 Departments of Neurology, Samsung Medical Center, Sungkyunkwan University School of Medicine, Seoul, Republic of Korea; 2 Translational and Stem Cell Research Laboratory on Stroke, Samsung Medical Center, Seoul, Republic of Korea; 3 Clinical Research Center, Samsung Biomedical Research Institute, Seoul, Republic of Korea; 4 Departments of Hemato-oncology, Samsung Medical Center, Sungkyunkwan University School of Medicine, Seoul, Republic of Korea; OSWALDO CRUZ FOUNDATION, BRAZIL

## Abstract

**Background:**

Cancer and stroke, which are known to be associated with one another, are the most common causes of death in the elderly. However, the pathomechanisms that lead to stroke in cancer patients are not well known. Circulating extracellular vesicles (EVs) play a role in cancer-associated thrombosis and tumor progression. Therefore, we hypothesized that cancer cell-derived EVs cause cancer-related coagulopathy resulting in ischemic stroke.

**Methods:**

Serum levels of D-dimer and EVs expressing markers for cancer cells (epithelial cell adhesion molecule [CD326]), tissue factor (TF [CD142]), endothelial cells (CD31+CD42b-), and platelets (CD62P) were measured using flow cytometry in (a) 155 patients with ischemic stroke and active cancer (116 − cancer-related, 39 − conventional stroke mechanisms), (b) 25 patients with ischemic stroke without cancer, (c) 32 cancer patients without stroke, and (d) 101 healthy subjects.

**Results:**

The levels of cancer cell-derived EVs correlated with the levels of D-dimer and TF+ EVs. The levels of cancer cell-derived EVs (CD326+ and CD326+CD142+) were higher in cancer-related stroke than in other groups (*P*<0.05 in all the cases). Path analysis showed that cancer cell-derived EVs are related to stroke via coagulopathy as measured by D-dimer levels. Poor correlation was observed between TF+ EV and D-dimer, and path analysis demonstrated that cancer cell-derived EVs may cause cancer-related coagulopathy independent of the levels of TF+ EVs.

**Conclusions:**

Our findings suggest that cancer cell-derived EVs mediate coagulopathy resulting in ischemic stroke via TF-independent mechanisms.

## Introduction

Cancer and ischemic stroke are two of the most common causes of death in the elderly and have been reported to be associated with one another.[1] [2] [3] The number of people living with cancer is increasing worldwide due to an increase in life expectancy globally and also due to the advances in cancer therapy. Nation-wide follow up studies showed that the risk of stroke is about 1.5-fold higher in the first few months after diagnosis of cancer.[4] [5] [6] [7] However, the pathomechanisms of stroke in cancer patients are not well known.

Extracellular vesicles (EVs) are small, spherical membrane fragments shed from the cell surface or secreted from the endosomal compartment and are reported to be involved in the pathogenesis of various diseases. Circulating EVs are reported to play a role in cancer progression, such as carcinogenesis/metastasis and neovascularization/tumor growth, and cancer-associated thrombosis, such as venous thromboembolism (VTE).[8] [9] [10] [11] Tissue factor (TF) is not only the primary cellular initiator of blood coagulation but also a modulator of angiogenesis and metastasis in cancer. Elevated tumor TF expression and increased circulating TF+ EVs were reported in patients with cancer and VTE, but there have been conflicting results concerning whether TF+ EVs is associated with VTE in patients with cancer.[10] [12] [13] [14]

We hypothesized that EVs from cancer cells cause coagulopathy resulting in cancer-related stroke. Thus, we tested the levels of circulating EVs expressing cancer cell surface markers and the levels of TF-bearing EVs. In addition, D-dimer levels were measured as a means to assess the presence of cancer-related coagulopathy in patients with cancer and ischemic stroke. We then performed path analysis to evaluate whether TF-bearing EVs mediate the association between cancer cell-derived EVs and cancer-related coagulopathy.

## Methods

This is a part of a trial of Optimal Anticoagulation Strategy In Stroke related to Cancer (OASIS-Cancer, ClinicalTrials.gov Identifier NCT02743052) study. The OASIS-Cancer study is an observational study to investigate the biological markers for intravascular coagulopathy causing stroke and for monitoring the effects of anticoagulation therapy, in patients with active cancer and ischemic cerebrovascular disease.

From July 2009 to October 2014, we prospectively studied consecutive patients with active cancers and acute ischemic stroke or transient ischemic attack. Patients were registered at the Samsung Medical Center, South Korea, and the criteria for patient selection were: (1) focal symptoms and relevant lesions as seen with diffusion-weighted imaging (DWI), (2) undergone diagnostic workups, including vascular and cardiologic studies, and (3) active cancer, excluding primary intracranial tumor. Cases with active cancer were defined as those who had: (1) been diagnosed with cancer within 6 months prior to enrollment, (2) received treatment for cancer within the previous 6 months, or (3) had recurrent or metastatic cancer.[15] The following patients were excluded from the study: (1) had not undergone magnetic resonance imaging or without relevant lesions observed on DWI, (2) serum D-dimer was not measured within 24 hours of the onset of stroke, (3) had undergone complete remission of cancer or only had a remote history of cancer, (4) with incomplete workups for stroke etiology (either vascular or cardiologic studies), and (5) had a history of recent surgery, myocardial infarction, deep vein thrombosis, or any signs of infectious or immunological diseases that may influence plasma D-dimer levels. The local institutional review boards approved of this study. This study has been approved by the Samsung Medical Center Institutional Review Board, and has been conducted according to the principles expressed in the Declaration of Helsinki. All participants or their guardians provided written informed consent for participation in this study.

### Workups and Patient Groups

Data on patient age, gender, and the presence of stroke risk factors including hypertension, diabetes mellitus, hyperlipidemia, atrial fibrillation, ischemic heart disease, and tobacco consumption were collected. Data related to stroke or cancer including clinical signs and symptoms, type of cancer and cancer pathology, the presence of systemic metastasis, and the time from cancer diagnosis to stroke onset were recorded. Routine laboratory data were collected for all patients (routine blood tests and coagulation studies, including prothrombin time, activated partial thromboplastin time, fibrinogen, and D-dimer). Hemostatic markers of prothrombotic tendency, including antiphospholipid antibodies (anticardiolipid antibody, lupus anticoagulants, and β2-glycoprotein-1 antibody) were measured in stroke patients younger than 50 years to exclude hypercoagulable state caused by other than cancer, but none of them showed positive results. In addition, all patients underwent electrocardiography, echocardiography, cardiac telemetry for at least 24 hours, and brain magnetic resonance imaging. Stroke mechanisms were assigned independently by two neurologists using criteria from the Stop Stroke Study Trial of Org 10172 in Acute Stroke Treatment (SSS-TOAST) and finalized by consensus.

Patients were classified into 2 groups according to the presence of conventional stroke mechanisms (CSMs), such as atherosclerosis, cardioembolism, and small-artery occlusion: (1) the CSM group (cancer-unrelated stroke group) and (2) the embolic stroke of undetermined source, except active cancer, group (cancer-related stroke group).[2] [16] In cancer patients without CSMs, a cancer-specific mechanism can be considered as the main cause of stroke. Our recent multicenter prospective study of 161 patients with active cancer who experienced acute ischemic stroke showed that stroke outside CSMs occurred in a large proportion (~40%) of cancer patients.[2] Coagulopathy with microembolism was more commonly observed in patients without CSMs than in those with CSMs (57.9% vs. 33.3%).[3] Cancer-specific mechanisms were unlikely to play a role in the development of stroke among patients exhibiting CSM, given that the distribution of stroke subtype among cancer patients with CSM was similar to that in stroke patients without cancer.[2]

Most cancer-related stroke patients received anticoagulants (93 of 116 patients, 80.2%), including intravenous heparin (n = 36), subcutaneous low molecular heparin (n = 47), or oral anticoagulants: either warfarin (n = 6) or non-vitamin K oral antagonists (n = 4). Patients were recommended to receive available cancer treatment (e.g., chemotherapy or radiotherapy). The D-dimer and EV levels were then measured serially, before (2.8 ± 3.9 days after symptom onset) and after treatment of anticoagulation (3.9 ± 2.8 days).

Patients with locally advanced or metastatic lung cancer (mostly adenocarcinoma) without a history of stroke were recruited as controls for the cancer group, because cancer-related coagulopathy is particularly associated with this type of cancer. In addition, patients admitted for ischemic stroke (without a history of cancer) at the Stroke Center at Samsung Medical Center between September 2014 and October 2014 were recruited as controls for the stroke group. Healthy subjects without a history of stroke or cancer served as a negative control group. This study has been approved by the Samsung Medical Center Institutional Review Board, and has been conducted according to the principles expressed in the Declaration of Helsinki.

### Measurement of Circulating EVs and Flow Cytometry

The expression of epithelial cell adhesion molecule (EpCAM, also known as CD326) and TF (also known as CD142) on the surface of circulating EVs was evaluated by flow cytometry. EpCAM a cell surface marker is highly expressed in a variety of carcinomas and is a prognostic marker in various carcinomas.[17] [18] [19] In addition, EpCAM is also a target of antibody-based therapies. The levels of endothelial and platelet surface markers (CD31+CD42b- and 62P) on EVs were also evaluated. This method allowed fractionation of specific populations of EVs based on the expression of cancer cell markers and TF, adjusted for total numbers of endothelial or platelet EVs (CD31+, a cell adhesion molecule on platelet or endothelial cells), such as: CD326+/CD31+, CD326+CD142+/CD31+, or CD142+/CD31+.

Citrated whole blood was collected and centrifuged at 1800 *g* for 15 min to obtain platelet-poor plasma. Plasma (250 μL) was thawed and centrifuged for 10 min at 19800 × *g* at 10°C to collect the EVs.[20] The EV pellet was resuspended in 20 μL of phosphate buffered saline (PBS). EVs (5 μL) were then incubated with fluorescent monoclonal antibodies (5 μL each): phycoerythrin (PE)-labeled anti-CD31 (555446; BD Biosciences, San Jose, CA), allophycocyanin (APC)–labeled anti-CD42b (551061; BD Biosciences), APC-labeled anti-annexin V (AV; 550475; BD Biosciences), PE-labeled anti-CD62P (P-selectin; 55524; BD Biosciences), fluorescein isothiocyanate (FITC)-labeled anti-CD142 (4508CJ; American Diagnostica, Stamford, CT), and PE-labeled anti-CD326 (130-091-253, Miltenyi Biotec, Seoul, South Korea), PE—labeled anti-CD133(130-080-081, Miltenyi Biotec, headquarters, Bergisch Gladbach, Germany).

The samples were incubated in the dark for 15 min at room temperature. Following the incubation 400 μL 1× binding buffer was added to the samples and a FACS Calibur flow cytometer using the CellQuest software (BD Biosciences) was used to collect and analyze the data. EVs were analyzed using a protocol with both forward scatter (FSC) and side scatter (SSC) in logarithmic mode and 10000 events were acquired for each sample. EV levels were normalized for an isotype control antibody and dot plots were normalized by control antibody. Based on the number of events (N) in the upper right (CD326-positive and CD142-positive) quadrant of the flow cytometric analysis (FL-2 vs. FL-4, corrected for isotype control antibody binding and autofluorescence) the number of EVs per liter of plasma was calculated as: n/L = N × (30/5) × (450/V) × (10^6^/250), where 5 (μL) is the volume of EV suspension, 30 is the total volume of washed EV suspension, 450 is the total volume in the tube before analysis, V is the sample volume analyzed, 10^6^ is the number of microliters per liter, and 250 μL is the original volume of plasma.[20] Standard beads 1.0 μm in diameter (Sigma; Molecular Probes, Eugene, OR, USA) were used for estimation of the EV size and EVs smaller than 1 μm were quantified. Laboratory personnel who conducted the blood assays were blinded to the subject’s clinical or laboratory data.

**Fig 1:**
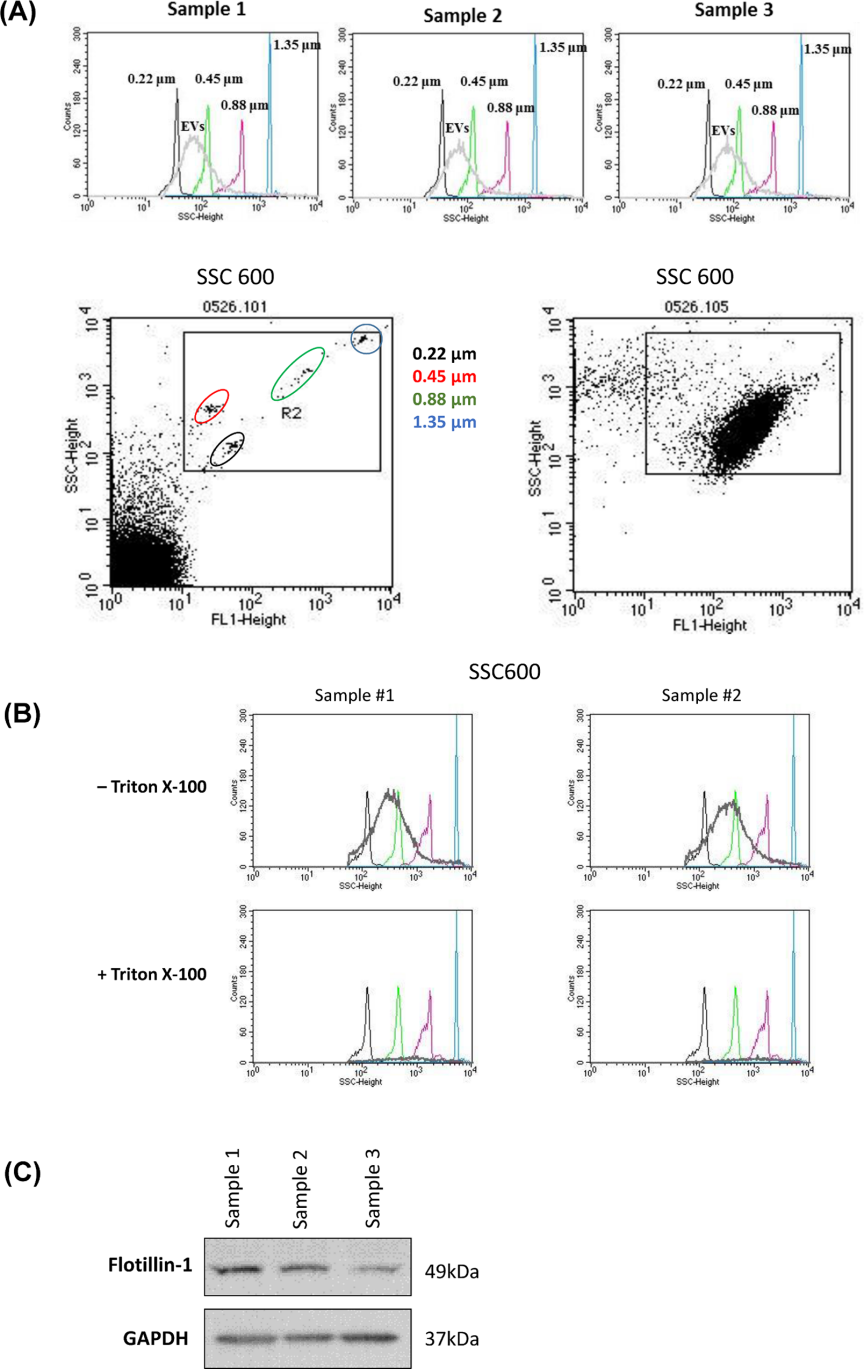
Flow cytometry and Western blot test results. (A) Flow cytometry results using size beads showed that most circulating cancer-derived extracellular vesicles (EVs) were distributed with a size between 200nm-1,000 nm. (B) Most EVs were degradated after treatment with 0.1% triton-X100. (C) Western blot test showed that EVs expressed flotillin-1.

In order to confirm the size distribution of EVs, fluorescence conjugated size beads (Nano fluorescent size standard, Spherotech, Lake Forest, IL) were used. Flow cytometry data showed that most EVs were distributed with a size between 200 nm and 1,000 nm on our SSC voltage setting (Fig 1A). In addition, we mixed 0.1% triton-X100 with EVs to distinguish between EVs and immunocoplexes (ICs) or protein aggregates. The vesicular structures are more sensitive to detergent compared to ICs and protein aggregates.[21] Most EVs were degradated after treatment with 0.1% triton-X100, precluding the possibility of contamination of ICs or protein aggregates. EVs expressed flotillin-1, a lipid raft associated molecules, which was used as EV marker (Fig 1B and 1C).[22]

The coagulation status was assessed based on the serum D-dimer levels. Levels of D-dimer and EVs were measured serially in patients with ischemic stroke and active cancer, before and after anticoagulation treatment.

### Statistical Analyses

Differences in discrete variables among the groups were evaluated using the chi-square test, Fisher’s exact test, or the Mann-Whitney test. Differences in continuous variables were analyzed using the one-way analysis of variance, the Kruskal-Wallis test, or the t-tests. The levels of cancer cell-derived EVs, D-dimer, and TF-bearing EVs were compared among the groups. Spearman’s correlation analysis was used to test the relationship between the levels of circulating EVs expressing cancer surface markers and TF-bearing EVs and D-dimer levels. Path analysis was performed to evaluate whether alteration of TF-bearing EVs mediates the effects of cancer cell-derived EVs on the development of coagulopathy and resulting cancer-related stroke. Path analysis is a method for studying direct and indirect effects. The aim of path analysis is an explanation, not a prediction. It is a causal modeling approach to exploring the correlations within a defined network, and is used to describe the directed dependencies among a set of variables. In this study, exogenous variables (cancer cell-derived EVs) were modeled as having both direct and indirect effects through an endogenous variable 1 (TF-bearing EVs) on dependent variables (D-dimer, an endogenous variable 2). In all analyses *P*<0.05 was considered statistically significant. Commercially available software (STATA, version 13.1; Stata Corp, College Station, TX, USA) was used for statistical analyses.

## Results

A total of 313 patients were enrolled in this study: 155 patients with ischemic stroke and active cancer (117 − cancer-related, 38 − CSMs), 25 patients with ischemic stroke but no cancer (stroke control group), 32 active cancer patients without ischemic stroke (cancer control group), and 101 healthy subjects (healthy control group) (Table 1). The mean patient age was 67.2 (standard deviation, 11.4; full range, 26–94 years) out of which 158 were men and 155 were women. The cancer control group had similar characteristics in terms of demographics, vascular risk factors, antithrombotic use, and chemotherapy compared to the patients with ischemic stroke and active cancer (*P*>0.05 in all cases). The risk factors for stroke, such as hypertension and hyperlipidemia, were more prevalent in the cancer-unrelated stroke group than in the cancer-related stroke group (*P*<0.05 in all cases) (Table 1).

**Table 1:** Patient groups

*Characteristics*	Stroke and active cancer		Control group		
	Cancer-related	Conventional stroke mechanisms	Stroke alone	Cancer alone	Healthy subjects
No, of patients (samples)	116 (233*)	39 (62)	25 (44)	32 (52)	101 (101)
Age, year (SD)	65.2 (9.9)	66.3 (9.5)	71.5 (11.0)	57.9 (10.5)	57.2 (12.2)
Male gender, No. (%)	59 (50.9)	30 (76.9)	12 (48.0)	17 (53.1)	46 (45.5)
Risk factors, No. (%)					
Hypertension	44 (37.9)	21 (53.8)	18 (72.0)	8 (25.0)	30 (29.7)
Diabetes mellitus	19 (16.4)	12 (30.8)	10 (40.0)	4 (12.5)	10 (9.9)
Hyperlipidemia	5 (4.3)	12 (30.8)	12 (48.0)	1 (3.1)	21 (20.8)
Atrial fibrillation	-	10 (25.6)	12 (48.0)	1 (3.1)	2 (2.0)
Cancer profiles, primary, No. (%)					
Lung	56 (48.3%)	12 (30.8%)	N/A	32 (100%)	N/A
Gastrointestinal	15 (12.9%)	8 (20.5%)	N/A		N/A
Hepatobiliary	24 (20.7%)	10 (25.6%)	N/A		N/A
Breast-gynecologic	12 (10.3%)	3 (7.7%)	N/A		N/A
Others	9 (7.8%)	6 (15.4%)	N/A		N/A
Total	313 (492)				

* Two follow up samples were obtained after patients determined to be cancer free were excluded from this study

N/A: not applicable

### The Levels of D-Dimer and Cancer-Cell-Derived EVs in Various Groups

**Fig 2:**
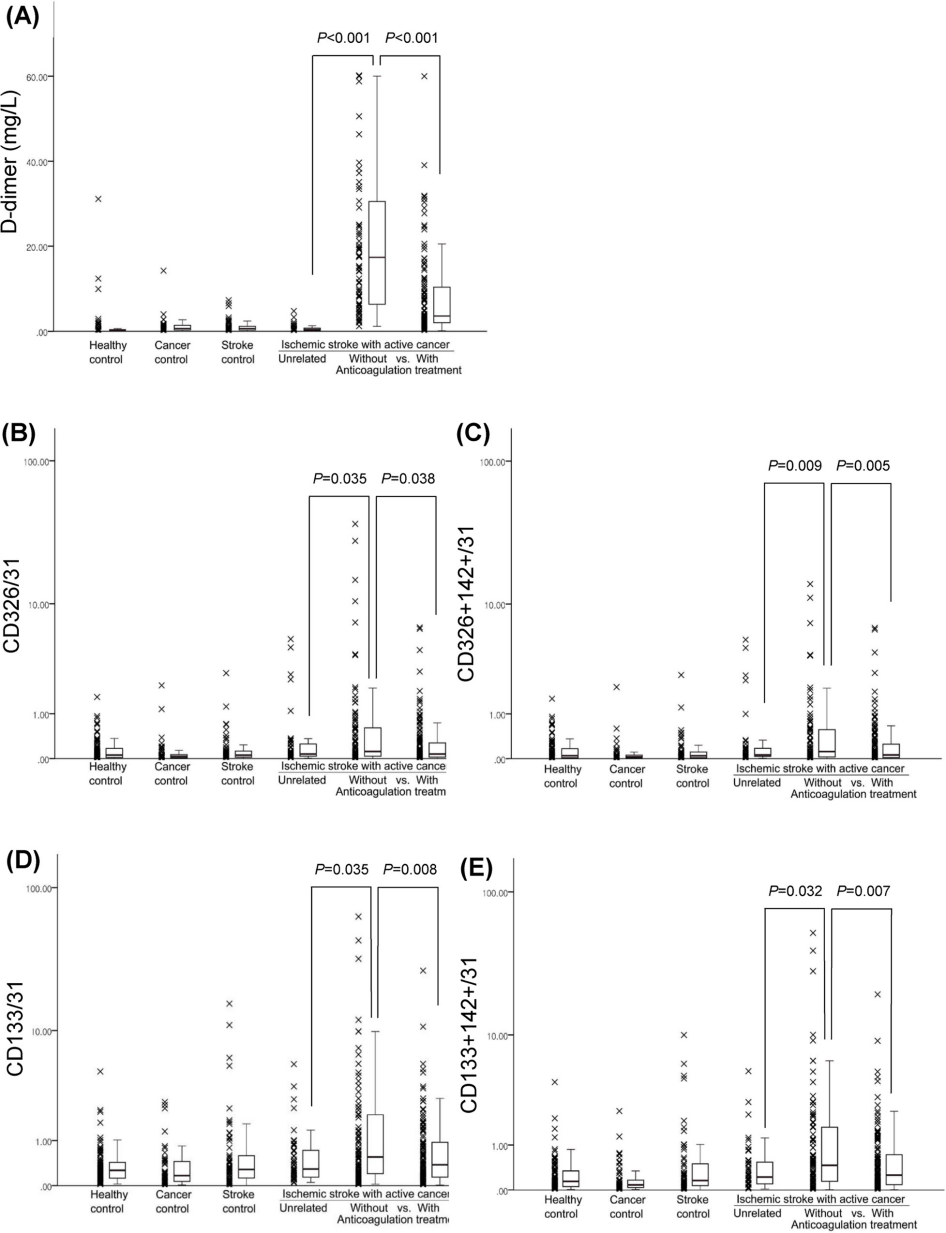
D-dimer levels (A) and cancer-cell derived EV levels (B–E) among the groups.

The D-dimer level was higher in patients of the cancer-related stroke group than in those of other groups (*P*<0.001). The levels of cancer cell-derived EVs (CD326+/CD31+ and CD133+/CD31+) and TF+ cancer cell-derived EVs (CD326+142+/CD31+ and CD133+CD142+/CD31+) were higher in patients with cancer-related stroke than in patients with cancer-unrelated stroke (*P*<0.05 in all cases) (Fig 2).

Cancer cell-derived EV levels correlated with levels of D-dimer (rho = 0.195, *P*<0.001) and with TF+ EVs (both cancer cell-derived [rho = 0.999, *P*<0.001] and platelet-derived EVs [rho = 0.427, *P*<0.001]).

D-dimer levels as well as levels of cancer cell-derived EVs and TF+ cancer cell-derived EVs showed a significantly decrease upon treatment with anticoagulation therapy (*P*<0.05 in all the cases) (Fig 2).

### Results from Path Analysis of Data

**Fig 3:**
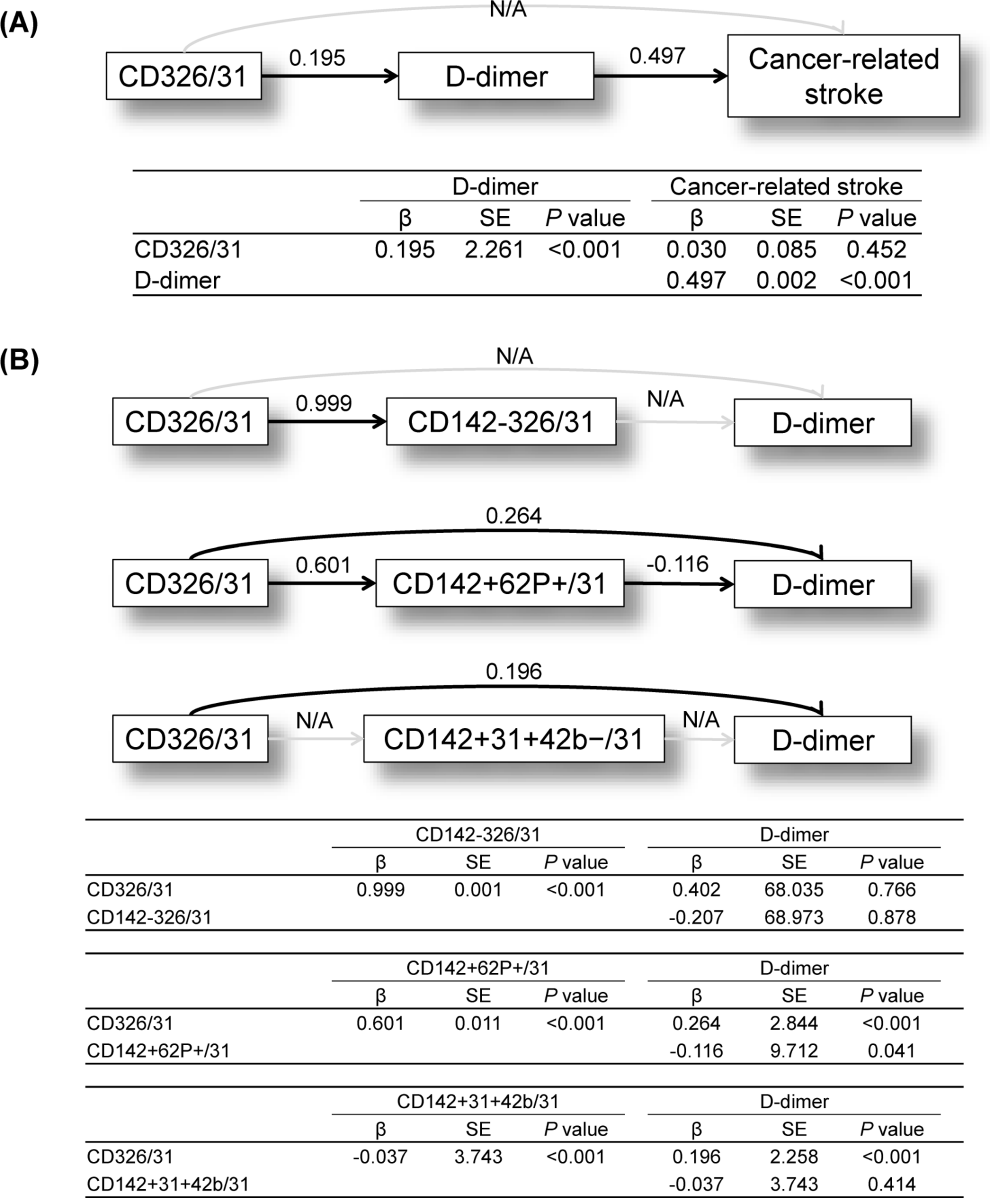
Results of Path analyses. (A) Path analysis using cancer-related stroke as the outcome, cancer cell-derived EVs as a predictor, and cancer-related coagulopathy as measured by D-dimer levels as a mediator. (B) Path analysis for cancer cell-derived EVs and cancer-related coagulopathy with tissue factor-bearing EVs (cancer cell-derived EVs, platelet-derived EVs, and endothelial-derived EVs) as mediators. N/A, no association. Numbers are β-coefficients that were statistically significant.

Since cancer cell-derived EVs and TF+ EVs were elevated in patients with cancer-related stroke therefore, path analysis was performed to evaluate the effect of cancer cell-derived EVs and TF+ EVs on coagulopathy and cancer-related stroke. Path analysis revealed that cancer cell-derived EVs are related to cancer-related coagulopathy (as measured by D-dimer levels) and cancer-related stroke (Fig 3A).

To test whether TF+ EVs mediate cancer-related coagulopathy, data from TF+ EVs derived from various tissues (cancer cells, platelets, and endothelial cells) were analyzed. Path analysis showed that cancer cell-derived EVs are related to coagulopathy via TF-independent mechanisms. While circulating cancer cell-derived EVs are associated with elevated levels of endothelial as well as platelet derived TF+ EVs however, these TF+ EVs are not related to coagulopathy: as measured by D-dimer levels (Fig 3B).

## Discussion

The main findings of this study are (a) cancer cell-derived circulating EVs are associated with coagulopathy resulting in stroke, and (b) this effect is mediated via TF-independent mechanisms.

### Cancer-Related Stroke

The characteristics of cancer-related stroke are distinct from those of conventional stroke. We have shown that embolism caused by intravascular coagulopathy is the main mechanism underlying cancer-related stroke.[3] Using modern diagnostic evaluations to improve our understanding of the characteristics of stroke in cancer patients is essential for the correct management of these patients.[23] [24] In addition, work up for hidden malignancy in patients with embolic stroke of undetermined source and suspected coagulopathy sometimes reveals hidden malignancies.[25] [16]

Our multicenter study of stroke patients with active cancer showed that D-dimer levels and DWI imaging of lesion patterns may be helpful in the early identification of cancer-related stroke cases as well as in establishing potential preventive strategies for stroke.[2] Assessment of D-dimer level is a direct measure of activated coagulation. Identification of predictors or mediators of coagulopathy may improve our understanding of the pathomechanisms of cancer-related stroke and may also help in monitoring the effect of treatment in those patients. While there have been efforts to find risk factors and candidate biomarkers for VTE in cancer patients[26] however, relatively little information is available on risk factors and biomarkers in the field of cancer-related stroke.

### Previous Studies on the Role of TF-Bearing EVs in Cancer-Related Coagulopathy

Preclinical studies showed the prothrombotic role of TF+ EVs in various tumor models.[27] [28] [29] [30] Hron et al. and others reported that TF+ EVs are known to be associated with VTEs in patients with cancer.[13] [10] On the contrary, Thaler and colleagues measured the EV-associated TF activity and showed that EV-TF activity was not associated with future VTE but associated with mortality in patients with systemic cancer, suggesting that TF+ EV are simply epiphenomenon of disease progression.[14]

The source of TF-bearing EVs could be cancer cells, platelets, endothelium, and other tissues. One study comparing TF+ EVs in 20 patients with colorectal cancer and 20 control subjects showed that TF+ EVs levels were two-fold higher in cancer patients mainly due to a higher amount of platelet derived TF+ EVs. This increase correlated with D-dimer levels.[13] Recent publications suggest the importance of ‘the platelet-cancer loop’: a reciprocating loop in which tumor-induced platelet activation results in platelet-induced tumor growth and dissemination, which in turn acts as a thrombosis trigger/tumor amplifier.[31] Geddings and colleagues showed that both high and low levels of TF+ cancer cell-derived EVs activated platelets and induced thrombosis, and that cancer cell-derived EV-induced platelet activation was required for enhanced thrombosis.[32] Several strategies evaluated to inhibit the platelet-cancer loop include: antiplatelet agents for cancer prevention, use of inhibitors of P-selectin, and therapeutic removal of platelets from plasma.[33] [31] However, concerns about the efficiency and safety of these strategies are limiting factors in recommending these as therapeutic options for cancer patients.[31]

On the contrary, our present study demonstrates that cancer cell as well as platelet derived TF+ EVs are not associated with cancer-related coagulopathy. TF+ platelet-derived EV levels were elevated in all stroke patients regardless of the mechanisms leading to stroke (including stroke controls) suggesting that this elevation is secondary to the infarction and a not primary pathology in cancer-related coagulopathy. Notably, most of the previous studies compared TF+ EV levels in cancer patients with or without VTEs and these studies did not evaluate patients with VTEs due to cancer-unrelated conditions.

### Possible Mechanisms of EV-Mediated Coagulopathy via TF-Independent Pathway

The results of the present study suggest that both coagulopathy and ischemic stroke in cancer patients were correlated with cancer cell-derived EVs but not with TF+ EVs. There may be additional prothrombotic mechanisms underlying coagulopathy beside TF-dependent pathway.

Various substances secreted by cancer cells such as cysteine proteases and sialic acid moieties of mucin possess procoagulant properties and activate factor X and factor VII.[34] [35] It is also proposed that cancer cells can directly stimulate factor X.[36] Cancer procoagulant is mainly found in malignant tissue and is a 68 kDa cysteine protease that activates factor X directly: independent of factor VII.[37]

In addition, TF-independent mechanisms of thrombosis have recently been introduced. Leukocytosis could be responsible for the thrombotic state, and cancer induces a systemic environment that primes neutrophils to release neutrophil-extracellular traps (NETs). NETs were originally described as a defense mechanism against infection.[38] Recently, NETs have been associated with cancer-related thrombosis[39] and recently suggested to play a role in cancer-associated arterial microthrombosis, such as ischemic stroke.[40] Recently, TF-independent polyphosphate-dependent/factor XII-triggered coagulation mechanisms were reported to be associated with thrombosis in a prostate cancer model.[41]

Lastly, differential expression of circulating miRNAs has been reported in various types of cancers.[42] EVs and miRNAs have the ability to transfer biological information to recipient cells and play an important role in cancer metastasis and prognosis.[42] [43] Therefore, EVs and their cargo proteins and miRNAs could be a tool for cell-cell communication between cancer cells and platelets. Our group is therefore studying the role of EVs in coagulation activation *in vitro* and miRNA profiles of circulating EVs in patients with cancer-related stroke.

### Limitations and Conclusions

This study has several limitations. Firstly, cancer-mediated hypercoagulability is a complex process involving dysfunction of multiple factors besides EV formation, including platelet function, fibrinolysis, the coagulation cascade, and endothelial integrity. In this study, only platelet- and endothelial-derived EVs, and D-dimer were measured. In addition, using D-dimer levels alone to attribute a stroke mechanism to hypercoagulability may not be a definitive method.[44] [45] D-dimer levels are nonspecific and may change with treatment or complications such as infection. Other mechanisms of stroke such as tumor emboli or chemotherapy-related coagulopathy could also be the underlying mechanisms of stroke in patients with cancer-related stroke. However, we excluded patients with conditions which may influence plasma D-dimer levels. Secondly, although the results of the present study suggest that anticoagulant use may be helpful in regulating D-dimer levels as well as cancer cell-derived EVs, longitudinal long-term follow-up data is needed to prove or disprove our results. Thirdly, we have used EpCAM as a cancer cell marker in the present study however EpCAM, is a non-specific marker of cancer cells. Although EpCAM has traditionally been of significant interest for the diagnosis and therapy of various epithelial cancers, multiple other functions of this glycoprotein have recently been reported.[46] The level of EpCAM is high in proliferating cells. Epithelial proliferation leading to regeneration and repair is critical for tissue healing following various forms of tissue diseases including liver damage and renal reperfusion injury.[47] [48] EpCAM is also up regulated during inflammatory responses.[17] In the present study, the level of EVs expressing CD326 was increased in patients in the stroke control group as well as in patients with cancer-related stroke. Therefore, it is possible that this increase of the pan epithelial marker EpCAM in the stroke control group may be related to stroke per se. No studies have been performed to evaluate this phenomenon in stroke patients, and calls for future studies. However, our analysis of EVs expressing other cancer markers, such as CD133 (a cancer stem cell marker), also showed a significant correlation with D-dimer levels (rho = 0.150, *P* = 0.024 for CD133+/31+). Fourthly, the data evaluated here were from a unique population: Korean patients with cancer. Therefore, further investigations of different populations are warranted. Lastly, although path analysis is useful tool for analyzing multiple causalities, there are still several problems. Collinearity can occur when independent variables are highly correlated, and influence on the estimation of path coefficients to be less accurate. In addition, sample size needs to be large to ensure stable parameter estimates. Further studies with a larger cohort is needed. Nevertheless, our findings have implications for understanding how coagulopathy underlie the relationship between cancer cell-derived EVs and stroke.

### Conclusions

In conclusion, our data suggest that cancer cell-derived EVs mediate coagulopathy resulting in ischemic stroke via TF-independent pathways. Further studies are needed to elucidate the precise mechanisms by which cancer cell-derived EVs cause coagulopathy. In addition, the role that procoagulants and miRNAs from cancer cell-derived EVs play in the pathogenesis of TF-independent coagulopathy, needs to be evaluated.
